# Supplementary material for: Athlete perceptions of virtual reality and barriers to its use in sport: A qualitative examination
Source: PLoS One. 2025 Apr 29;20(4):e0320225. doi: 10.1371/journal.pone.0320225 (PMC12040243; doi:10.1371/journal.pone.0320225)
Supplement: S1 File — (DOCX) [file pone.0320225.s001.docx]

Athlete perceptions of virtual reality and barriers to its use in sport: A qualitative examination

Interview Guide

1. Hello, my name is _______ and I am from College of Applied Human Sciences at West Virginia University. In this project, I am talking to athletes about why, when, where, and how they have used virtual reality for sport training and what that training consists of. As a reminder, our conversation today will be recorded, and the information you share will be confidential to the extent permitted by law.
   1. First, I’m going to define VR for you:
      1. Definition of VR– Virtual reality is a visual‐based computer simulation that reproduces realistic and interactive 3D environments, including realistic images, sounds, and other sensations. In sport training, there are two key types of VR:
         1. Fully immersive VR: Uses head-mounted displays or a cave automatic virtual environment. Fully immersive VR produces high quality visualizations that are observed from the first‐person viewpoint and completely fill the user’s field of view.
         2. Semi-immersive VR: this type of VR engages a smaller field of view and allows the user to look “outside” the virtual environment. Scenery can be projected on big, flat, or cylindrical screens. This type of VR allows more freedom of movement than the head-mounted displays, such as whole body actions.
   2. VR can be used for a variety of tasks, and has been used in fields like medicine, counseling, and the military. Recently, it has also become more popular in sport. However, we are still learning about how it is being used for sport training.
      1. Do you have any questions about what I mean with VR? Is this similar to how you would define it?
      2. In this interview, we will be referring to times before, during, or after practice or a competitive event when you are using VR to train for your sport _____________ (interviewer to name sport). It may involve learning or practicing skills, preparing for an event, or competing.
2. Could you describe a time you used VR to train for your sport [wait for a response]?
3. 4 Ws questions
   1. I am interested in what type of VR you were using. What type of VR equipment have you used? (**What**).
   2. Where did your VR use take place? **(Where)**
   3. Describe your use of VR in the following situations **(Where and When)**
      1. Practice
         1. Could you describe how you use VR during practice?
      2. Pre-competition (night before or just prior to competition)
         1. Could you describe how you use VR before competition?
      3. During competition
         1. Could you describe how you use VR during a competition?
      4. Post competition
         1. Could you describe how you use VR after competition?
   4. What skills do you believe VR can be used to train? **(Why)**
      1. Probe: Have you used it to train you anticipation skills? Decision-making? Movement? Coping? Motivation?
      2. Probe: Do you believe that VR was effective in training those skills?
      3. Probe: Do you use VR for other purposes? If so, could you describe what you use it for?
4. TAM questions
   - 1. Did you find VR easy to use? Why or why not?
     2. Do you think VR is useful for your sport? If so, could you provide examples about how?
     3. Are you willing to use VR again in the future?
5. More specific questions and probes
   - 1. What was you experience like the first time you used VR
     2. Was there an acclimation period during your first VR use?
     3. When you use VR, how does it make you feel?
     4. When you use VR, what senses are part of this experience?
     5. How frequently have you used VR (in general)?
     6. How often do you use VR for sport training?
     7. How long do your training sessions typically last?
     8. Where do your training sessions typically take place?
     9. Have you ever used VR while recovering from an injury?
        1. If so, did you use it as part of the rehabilitation process? What did that look like?
     10. Is your displayed image or video from a camera perspective as if someone is taking a video of you or do you see the images from your own eyes?
     11. What sort of apps or videos do you use during VR training?
         1. Probe: are they custom made for you or mass produced?
         2. Do you use pre-made apps or video created specifically for you?
6. Do you think other athletes are aware of the potential of VR use for sport training?
   1. What about coaches?
7. What do you think are some barriers to more athletes using VR?
   1. How can we address those barriers?
8. Is there anything else about your VR experiences that you would like to share?
